# Performance of Widal test and stool culture in the diagnosis of typhoid fever among suspected patients in Dar es Salaam, Tanzania

**DOI:** 10.1186/s13104-019-4340-y

**Published:** 2019-06-05

**Authors:** Akili Mawazo, George M. Bwire, Mecky I. N. Matee

**Affiliations:** 1 Department of Microbiology and Immunology, School of Medicine, Muhimbili University of Health and Allied Sciences, P.O. Box 65001, Dar es Salaam, Tanzania; 2 Department of Pharmaceutical Microbiology, School of Pharmacy, Muhimbili University of Health and Allied Sciences, P.O. Box 65013, Dar es Salaam, Tanzania

**Keywords:** Blood culture, Dar es Salaam, Referral Hospital, Stool culture, Widal test

## Abstract

**Objective:**

We set an experiment to determine the diagnostic performance of the Widal test and stool culture in typhoid-suspected cases attending tertiary hospitals in Dar es Salaam, Tanzania using blood culture as a golden standard. We also evaluated the agreement between Widal, stool and blood culture.

**Results:**

This was a cross-sectional study conducted between June and September 2018, in three Regional Referral Hospitals in Dar es Salaam, Tanzania. A total of 158 typhoid-suspected cases were enrolled, after obtaining an informed consent. Of the 158 patients participated in the study, 128 (81%) tested positive for the Widal test and 17 (11%) patients were stool culture positive. Widal test recorded 81.5% sensitivity, 18.3% specificity, 10.1% positive predictive value and 89.7% negative predictive value. Stool culture showed 31.3% sensitivity, 91.5% specificity, 29% positive predictive value and 91.5% negative predictive value. In conclusion, Widal test is not reliable for diagnosis of typhoid fever since false positive and negative results are common. In addition, Widal test recorded poor agreement with the blood culture (kappa = 0.014, p < 0.05) while stool culture had strong agreement with the blood culture (kappa = 0.22, p < 0.05).

## Introduction

Typhoid fever is an infectious disease caused by *Salmonella typhi*. Less commonly, strains of non-typhoidal Salmonella usually cause intestinal infections associated with diarrhoea, fever, and abdominal cramps that often last 1-week or less commonly longer, non-typhoidal Salmonella can cause extra intestinal infections such as bacteraemia and urinary tract infection [1].

Precise timely diagnosis of typhoid fever in early stages aims at identifying aetiological agent and carriers who might serve as source of transmission during outbreak [2]. Blood, bone marrow and stool culture are the most reliable diagnostic methods, with bone marrow culture being gold standard for typhoid. Definitive diagnosis of typhoid requires isolation of *S. typhi* from blood or stool [3].

The sensitivity of blood/stool culture ranges from 40 to 97% if the patient has not used antibiotics [4]. Blood and stool cultures, are less frequently used in developing countries due to cost and requirement of highly trained professionals [5]. The most preferred method in regional, district and health centres in Tanzania where laboratory services are not advanced is Widal test, which is easy, cheaper, and does not need highly trained laboratory personnel.

Widal test has been associated with some controversies which include, inherent variabilities of the test, difficulty in establishing a steady-state baseline titre for the population, repeated exposures to *S. typhi* in endemic regions, cross-reactivity with other non-Salmonella organisms and lack of reproducibility of the test result [6]. The test also relies on the demonstration of a rising titre of antibodies in paired samples 10 to 14 days apart [7].

In typhoid fever, it is so difficulty to demonstrate such a rise even in blood culture proven patients [5]. It is also not practical as patients cannot be kept waiting without initiating treatment and in developing countries returning to hospital for second time is costly and is not practical when [8]. The Widal test is affected by other factors including cross reactivity of other salmonella subspecies, which are not the direct cause of fever and also may turn positive in malaria infection [4].

In developed countries, Widal test is no longer being used due low prevalence of typhoid fever, access to safe water, better laboratory techniques to isolate bacteria, and poor performance of the Widal test [5]. In contrast, most developing countries, including Tanzania, Widal test continue to be used in the diagnosis of typhoid fever, and are the second requested test after malaria tests [9, 10].

This study was conducted in the three-referral hospitals in Dar es Salaam, among patients, with clinician’s request for Widal test, to compare performance of the Widal test and stool culture using blood culture as reference test.

## Main text

### Methods

#### Study design and study area

This was a cross-sectional study conducted from June to September 2018 in Amana, Temeke and Kinondoni Regional Referral Hospitals in Dar es salaam, Tanzania. We consecutively enrolled 158 patients with Widal test requests from physicians after obtaining an informed verbal and written consent.

#### Study population

Patients aged 5–82 years attending clinic at Dar es Salaam Referral hospital suspected to have typhoid fever by their attending clinicians.

#### Sample collection and processing

Well-trained technologist collected 5–10 mls of venous blood samples aseptically depending on the age of participant. 3–8 mls depending on age were inoculated directly into the broth and 2–3 mls were collected in plain vacutainer tubes for widal.

A spoonful of stool sample was collected in clean containers.

#### Laboratory testing typhoid

##### Widal test

We used serum to detect specific O and H antigens. A drop of O antigens and H antigens was added in the test tubes, equal amount in all. Based on the manufacturer manual, an antibody titre of 1:80 and higher for anti TO and 1:160 and higher for anti TH antibodies was taken as a cut off value to indicate recent infection of typhoid fever.

##### Stool culture

We inoculated onto MacConkey agar plates and xylose, lysine deoxycholate and incubated at 37 °C for 48 h. We subjected to Gram stained suspected Salmonella colonies and presumptively identified using Kligler Iron Agar (Difco™), urease test (Himedia ltd. India), Indole, Oxidase Citrate, Motility.

##### Blood cultures

The broth was prepared from 10% ox-gall/Columbia agar—broth in distilled water. (Add 5 ml of whole blood to 50 ml of sterile ox-bile medium. After the overnight incubation, sub culturing and biochemical identification was performed from the 10% ox-gall/Columbia on XLD agar (OXOID, England).

In XLD agar: H_2_S producing salmonella formed pink red colonies of 3–5 mm in diameter with black centres.

In MacConkey agar: salmonella produced non-lactose fermenting pale coloured colonies. All these media and broth used will be prepared according to manufacturer’s instruction.

#### Biochemical identification

Salmonella was presumptively identified using Kligler Iron Agar, (Difco™), urease test (Himedia ltd. India), Indole, Oxidase Citrate, Motility.

#### Quality control

Standard operational procedures were followed during processing of each sample. All the instruments used for sample processing were checked every morning for proper functioning.

*E. coli* ATCC 25922 was used as a reference strain.

#### Data analysis and management

We crosschecked and coded Data on laboratory results before entering into computer software. Data were cleaned and analysed using Statistical Package for Social Sciences (SPSS) version 23.0 (SPSS software Chicago Inc., USA). Summarization of data was done using frequency distribution and two-way tables; the association between other independent and dependent variables was determined using Chi-square or kappa Test. The value of kappa, < 0.09; 0.1–0.19; 0.2–0.49; and > 0.5 were considered poor, moderate, strong and almost perfect agreement respectively. p value of < 0.05 was considered statistically significant.

### Results

#### Participants’ characteristics

We enrolled 158 patients. Median age was 34 years, mean 31 and females 62%. Participants had either secondary education 43.8% or lower level education 41.2%. Majority of the participants were either married 62 (40%) or divorced 42 (27.1%). Patients response on not using of antibiotics in 2 weeks, 1 week, 3 days respectively, 93 (58.5%), 47 (28.9%) 13 (8.2%). On the timing of 2 weeks, 1 week and 3 days respectively sickness response was 74 (46.4%), 47 (29.6%) and 7 (4.4%) (Table 1).

**Table 1:** Socio-demographic characteristics of study population

Variable	Frequency	Percentages
Median age (IQR)	25 (20–30)	
Age group		
< 25	50	31.6
25–40	59	37.8
40–60	31	19.6
≥ 60	18	11.4
Level of education		
Informal/none	11	7
Primary	54	34.2
Secondary	56	35.4
University/College	19	12
Others	18	11.4
Marital status		
Single	39	26.4
Married	62	39
Divorced	42	26.4
Widowed	12	7.5
Sex		
Male	60	37.7
Female	98	61.6

8 of 93 non-antibiotics user 2 weeks before hospital visit, 8 were blood culture positive. 1 of 13 antibiotic user 1 week before coming to hospital was blood culture positive and 2 of 46 who used antibiotics in 2 weeks before coming to hospital were blood culture positive.

The mean (standard deviation) duration of sickness was 6 (4). Among 74 patients who fell sick within 1 week, only eight were blood culture positive, 47 in 2 weeks only six were positive and among 25 who started feeling sick in 3 weeks only 3 were blood culture positive.

#### Sensitivity, specificity and predictive values of Widal and stool culture in the diagnosing typhoid fever using blood culture as a standard method

Out of the 158 blood specimens cultured, 16 (10.1%) were positive for *S. typhi*. Thus, the overall prevalence of typhoid fever in the study population was 10.1%. The prevalence according Widal was 80.5% while prevalence according to stool culture was 11% where isolates in the stool were not serotyped (Table 2).

**Table 2:** Sensitivity, specificity and predictive values of Widal and stool culture in the diagnosing typhoid fever using blood culture as a standard method

Test	Sensitivity (%)	Specificity (%)	PPV (%)	NPV (%)	Kappa test
Stool	31.3	91.5	29.4	92.2	0.222
Widal	81.2	18.3	10.1	89.7	0.01

Compared against blood culture, stool culture had sensitivity, specificity, positive predictive value (PPV) and negative predictive value (NPV) of 31.25%, 91, 5%, 29.4% and 92.2%, respectively while Widal test had sensitivity, specificity, PPV and NPV of 81.5%, 18.3%, 10.1% and 89.6%, respectively (Table 2).

### Discussion

As the world continue fighting against antimicrobial resistance, correct, rapid and accurate diagnosis is needed toward archiving the goal. In Tanzania, febrile presenting diseases such typhoid fever are among the disease which are commonly diagnosed [10]. Therefore an experiment was done to evaluate the diagnostic accuracy of the commonly performed Widal test and stool culture while keeping blood culture as a golden standard.

The actual prevalence of typhoid fever was found to be 10.1%, meaning for every 10 patient who are tested positive, and only one was truly suffering from typhoid. The prevalence reported in this study has also been reported in other studies conducted in Cameroon [7].

The consequences of false positive results include misuse of antibiotics, likely danger of increasing the antibiotic resistance, increased cost of treatment due increased hospital stay for inpatients and missing of fatal disease of febrile illnesses.

The sensitivity, specificity, PPV and NPV of Widal titration in this study was 81.2% 18.3%, 10.1 and 89.7 respectively. Widal test had a fair sensitivity and negative predictive value; it also had very low specificity and positive predictive value. This might be due to cross-reacting antibodies from other enterobacteria [11]. Low specificity means patients might be suffering from other causes of diseases and low PPV value means majority of those who tested to have disease are not truly diseased by the suspected cause.

A systematic review found the mean sensitivity, specificity, PPV and NPV of 73.5 with SD 12.6 minimum of 45.2 and maximum of 98, 75.7 with SD of 20.2, minimum 13.8 and maximum of 98, 75.2 with SD 24.8 minimum of 31 maximum of 100 and 60 with SD of 29 minimum of 5.7 and maximum of 91 respectively [5]. The findings of this study are within range of systematic review.

Studies in Tanzania, Mtove in Teule Hospital Muheza found, sensitivity, specificity, NPV and PPV 75%, 98%, 100%, and 26% respectively. All these study concur with the findings of the review [5]. The study found, stool culture had sensitivity specificity, PPV and NPV 31.3, 91.5, 29 and 92.2 respectively. Low sensitivity may be associated with the sample collection time. Stool is not sensitive in early infection [11]. Sensitivity can be increased when specimen is taken in 3rd week. In stool culture, growth of Salmonella does not mean infection as the method can be used in identifying carriers. It is positive in 35% of cases in early infections [12].

Systematic review [11], stool culture had sensitivity, specificity, PPV and NPV of 71.4%, 66.7, 83 and 50 respectively. These findings correlate with the findings from the current study. This is due being prone to contamination that may result in misdiagnosis.

Comparing tests, stool performed better in terms of specificity and negative predictive value. Stool culture had good NPV of 92.2 and specificity of 91.5% while Widal test had specificity of 18.3 and NPV of 89.7%. On the other hand, Widal performed better in terms of sensitivity compared to stool culture. Widal test had sensitivity of 81.2 and stool culture has sensitivity of 31.3%. This means Widal test can be useful in excluding disease free cases and stool can be used in confirming suspected cases.

Statistically, there was a fair agreement (kappa = 0.33) between stool culture and blood culture, poor agreement (kappa = 0.01) between Widal titration and stool culture. Similar finding were observed in a study conducted by Abebe and his colleague [7]. Current finding disagrees with a study conducted in Addis Ababa, which indicated moderate agreement (kappa = 0.41) between Widal test and blood culture. Another study in Ethiopia got the finding, which disagree with this study, agreement between Widal tube agglutination test (titration) and stool culture was 0.33 (kappa = 0.32) while this study found very small agreement. This indicates that the result of Widal test in diagnosis of typhoid fever less likely agrees with stool culture.

Nakhla et al. did a study to find the test that can replace Widal test. She compared latex agglutination Dri-Dot assay and IgM lateral flow assay, to blood culture. Sensitivity of the Dri-Dot was 71.4%, and specificity was 86.3% for samples collected at time of first diagnosis. Sensitivity and specificity of IgM lateral flow were 80% and 71.4%, respectively although there was increased sensitivity to 84.3%, but decreased specificity to 70.5% when both rapid tests were performed in parallel [12].

### Conclusion

From the study, Widal test has a low specificity and PPV, but it has high sensitivity and good NPV with stool culture. Using single Widal test as the only laboratory test for the diagnosis of typhoid fever will produce wrong diagnosis. Widal test has poor agreement with the blood culture and stool culture; stool culture has good agreement with the blood culture. This means Widal test should not be used alone but in combination with blood/stool culture test.

The high NPV value meant that Widal test could only be useful for knowing non-diseased in the population. The low PPV value meant that Widal test could only be useful for excluding the diseases from the population. Stool culture had high sensitivity a specificity and PPV, stool culture performed better than Widal test.

## Limitations

The Widal false negative results can be probably because blood was collected in early disease processes. Previous antibiotic treatment may also contribute to negative blood culture test. The original Widal agglutination test was described using paired sera obtained 10 days to 2 weeks apart.

## Data Availability

All relevant data generated and analysed during this study are available from the corresponding author on reasonable request.

## Abbreviations

CA: Chrome Agar
CFU: colony forming unit
CLSI: Clinical Laboratory Standards Institute
DCA: Deoxycholate Citrate Agar
GNB: gram negative bacteria
KIA: Kligler Iron Agar
SSA: Salmonella–Shigella Agar
TSI: Triple Sugar Iron Agar
WBC: white blood cells
XLD: xylose, lysine and deoxycholate
